# Infection with hepatitis viruses, FIB-4 index and risk of hepatocellular carcinoma in southern Italy: a population-based cohort study

**DOI:** 10.1186/s13027-016-0101-x

**Published:** 2016-11-03

**Authors:** Mario Fusco, Pierluca Piselli, Saverio Virdone, Pietro Di Cicco, Paola Scognamiglio, Paolo De Paoli, Valerio Ciullo, Diana Verdirosi, Michele D’Orazio, Luigino Dal Maso, Enrico Girardi, Silvia Franceschi, Diego Serraino

**Affiliations:** 1 Registro Tumori, ASL Napoli-3 Sud, Brusciano, Napoli Italy; 2 Clinical Epidemiology Unit, National Institute for Infectious Diseases “L. Spallanzani”, Rome, Italy; 3 Unit of Cancer Epidemiology, CRO Aviano National Cancer Institute, Via F. Gallini 2, 33081 Aviano, (PN) Italy; 4 Azienda Sanitaria Locale (ASL) Napoli 3, Brusciano, Napoli Italy; 5 Scientific Directorate, CRO Aviano National Cancer Institute, Via F. Gallini 2, 33081 Aviano, (PN) Italy; 6 International Agency for Research on Cancer, Lyon, France

**Keywords:** Liver diseases, Relative risk, Epidemiology, Viral hepatitis, Liver fibrosis index, Southern Italy

## Abstract

**Background:**

The incidence of hepatocellular carcinoma (HCC) and its association with hepatitis C (HCV) and hepatitis B virus (HBV) infections, FIB-4 index and liver enzymes was assessed in an area of the province of Naples covered by a population-based cancer registry.

**Methods:**

We conducted a cohort investigation on 4492 individuals previously enrolled in a population-based seroprevalent survey on HCV and HBV infections. The diagnosis of HCC was assessed through a record linkage with the cancer registry. Hepatic metabolic activity was measured through serum alanine transaminase, aspartate aminotransferase, gamma-glutamyl-transferase, and platelet. The FIB-4 index was used as a marker of fibrosis. We computed HCC incidence rates (IR) for 100,000 (10^5^) person-years of observation, and multivariable hazard ratios (HR) with 95 % confidence intervals (CI) to assess risk factors for HCC.

**Results:**

Twenty two cases of HCC were diagnosed during follow-up (IR = 63.3 cases/10^5^). Significantly increased HCC risks were documented in individuals with higher than normal liver enzymes and low platelet count; in the 239 HCV RNA-positives (HR = 61.8, 95 % CI:13.3–286); and in the 95 HBsAg-positives (HR = 75.0) –as compared to uninfected individuals. The highest FIB-4 score was associated with a 17.6-fold increased HCC risk.

**Conclusions:**

An elevated FIB-4 index turned out to be an important predictor of HCC occurrence. Although the standard method to assess hepatic fibrosis in chronic hepatitis remains the histologic staging of liver biopsy specimen, the assessment of FIB-4 in HCV RNA-positive individuals may help in identifying the highest HCC-risk individuals who need anti-HCV treatment most urgently.

## Background

Hepatocellular carcinoma (HCC) - the fifth most common cancer in men (554,000 cases, 7.5 % of the total burden of cancer) and the ninth in women (228,000 cases, 3.4 %) worldwide - represents an important public health issue. The HCC burden is particularly heavy in less developed countries, where 83 % of the estimated 782,000 new HCC cases worldwide were diagnosed in 2012 [1]. Within the World Health Organization (WHO) European region, one of the highest HCC incidence rates is registered in southern Italy, mostly because of an epidemic in the Campania region, with yearly rates as high as 48.3 HCC cases/100,000 in men and 18.4/100,000 in women [2–4].

Infection with hepatitis C virus (HCV) and/or hepatitis B virus (HBV) are major risk factors for HCC [5, 6]. Worldwide, the estimated prevalence of HCV infection is about 2.2 %, with regional variations ranging from <1.0 % in northern Europe to >3 % in northern Africa. Within Europe, the lowest prevalence of HCV infection was reported in the United Kingdom and Scandinavia (0.01–0.1 %), and the highest one in southern Italy (7.5 %) [6].

In Italy, population-based sero surveys showed a strong North–south geographic gradient [7–10], with high prevalence of HCV in the southern part of the country. In this area, particularly elevated HCV prevalence rates were recorded among the older segment of the population (23 % of people aged 65 or more was HCV-positive in a population-based investigation conducted by our study group between 2003 and 2006) [11, 12]. Chronic HCV infection (i.e., HCV RNA–positivity) is more common than chronic HBV infection, and high HCV prevalence is mainly due to iatrogenic transmission in the past decades [12].

An estimated 55–85 % of people with HCV infection progress to chronic HCV infection, a condition that puts these people at risk of liver cirrhosis, liver failure or HCC at varying rates according to factors such as age, male sex, presence of other risk factors (e.g., alcohol), or to viral infections and metabolic characteristics [5, 6, 13, 14]. Liver fibrosis is a major negative prognostic marker, and the degree of histologic fibrosis in a liver biopsy sample is still the most common tool for staging liver diseases associated with HCV and/or HBV infections. However, such diagnostic procedure is affected by several drawbacks, and non-invasive serum markers have been proposed and validated to predict the progression of liver fibrosis, including the FIB-4 index -described in the Methods section [15–17].

In this study, we took advantage of a population-based cohort whose baseline HBV and HCV seroprevalence was evaluated between 2003 and 2006 in the province of Naples, an area of southern Italy covered by a population-based cancer registry and with high incidence rates of HCC [11]. The main aims of this analysis were to estimate incidence and determinants of newly diagnosed HCC cases among the cohort members with special emphasis on the consequences of HCV RNA-positivity- and the role of FIB-4 as an index of hepatic fibrosis in HCC.

## Methods

### Study design and population

The design of this population-based investigation is longitudinal. Our study took place in an area of the province of Naples, southern Italy, with about 650,000 inhabitants who are covered by a population-based cancer registry – the Campania Cancer Registry (CCR).

The enrollment in this population-based seroprevalence survey took place in 2003–2006, when 4496 individuals aged 20 years or older were randomly selected (after stratification for sex and age) from the resident population [11]. At enrolment, study participants were interviewed, blood sample taken and tested for HCV and HBV infections and for hepatic metabolic activity. Among these 4496 individuals, 336 were anti-HCV positives and 100 were HBsAg-positives, and they were the main target of this follow-up analysis.

### Inclusion criteria

For the aims of this cohort analysis, we excluded four of the 4496 individuals tested in 2003–2006 because they had been already diagnosed with HCC at enrolment (i.e., they were prevalent HCC cases).

### Data collection

A semi-automated, anonymous record linkage was carried out in October 2014 by means of a previously validated record linkage procedure [18]. We linked the database of the seroprevalence study and the database of the population-based CCR. HCC at CCR were identified according to International Classification of Diseases, 10th revision [19] and International Classification of Diseases for Oncology (ICDO, C22.0-C22.9 codes) [20]. Individual data regarding the occurrence of new HCC diagnoses and vital status were updated to December 2012 (last date of cancer registration).

Serum alanine transaminase (ALT), aspartate aminotransferase (AST), gamma-glutamyl transferase (γGT), and platelets counts were measured at enrolment with upper normal limits (UNL) of 40 U/L for AST and ALT; and 30 U/L for γGT [11].

To assess liver fibrosis, we used the FIB-4 index, computed according to the following formula [15]: FIB-4 = age [years] × AST [U/L]/(platelets [10^9^/L] × √ALT [U/L]). The FIB-4 values were divided into three groups/tertiles in order to make our results comparable with those already described in the literature [15–17]: FIB-4 <1.45, the lowest tertile, which identifies subjects without liver fibrosis; FIB-4 between 1.45 and 3.25, the intermediate tertile, which identifies subjects with mild to moderate fibrosis; and FIB-4 >3.25, the highest tertile, which identifies subjects with severe fibrosis.

**Fig. 1:**
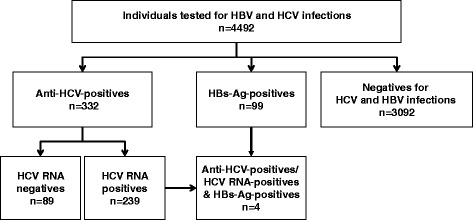
Distribution of cohort members according to HCV and HBV test results

Among the 4492 individuals included in this cohort analysis, 99 were chronically infected with HBV (i.e., they were HBsAg-positives); 243 were chronically infected with HCV (i.e., they were HCV RNA-positives); and 89 were anti HCV-positives but HCV RNA-negatives. Four individuals HBsAg-positives were also HCV RNA-positives (Fig. 1).

### Statistical analysis

Person-years (PY) at risk of HCC were computed from date of testing to date of HCC diagnosis; date of death or December 31, 2012, whichever occurred first. Incidence rates (IR) of HCC were computed by dividing the number of new cancers by PY at risk. The association between selected variables, collected at time of testing, and the occurrence of HCC at follow-up was estimated by using the Cox multivariable regression analysis through the calculation of hazard ratios (HR) and 95 % CIs adjusted for sex, age, HBV and HCV infection. The probability of surviving according to HCV infection was computed according to the Kaplan-Meier method [21].

All statistical analyses were performed using commercially available software (SAS Institute Inc., version 9.4, Cary, NC, USA; and STATA version 13, StataCorp LP, College Station, Texas).

## Results

The 4492 cohort members included in this study (44.7 % men) had a median age of 45.7 years (interquartile range -IQR: 30.5–60.3 years), and they had been followed up for a median period of 8.0 years (IQR: 7.2–8.6 years). The median time from testing to HCC diagnosis was 37 months, while the median age at HCC diagnosis was 73.3 years (data not shown).

Twenty-two cohort members developed HCC during the 34,749 PYs of follow-up: two were anti HCV-negatives and HBsAg-negatives; 15 were HCV RNA-positives; three were anti HCV-negatives and HBsAg-positives; and two were anti HCV-positives/HCV RNA-negatives and HBsAg-negatives.

Table 1 shows IRs and multivariate HRs for HCC according to selected characteristics collected at baseline. Overall, the IR of HCC was 63.3 cases/10^5^ PY, and the cumulative incidence at the end of the follow-up period was 0.5 % (22/4492). Elevated HCC IRs were recorded among subjects aged 60 years or older (252.8/10^5^ PY in those ≥70 years), in HCV RNA-positives/HBsAg-negatives (909.3/10^5^ PY), and in HBsAg-positive/HCV-negative individuals (403.3/10^5^ PY). IRs of HCC were highly increased in cohort members with liver markers exceeding by three or more times the normal level, i.e., 1598 HCC cases/10^5^ PY for ALT; 3539 cases/10^5^ PY for AST; and 1006 cases/10^5^ PY for γGT. Similarly, individuals with the highest tertile of FIB-4 index (i.e., >3.25) showed a very high IR of HCC, 1889 cases/10^5^ PY (Table 1). Eighty-nine anti HCV-positive/HCV RNA-negative individuals developed two HCC cases (IR = 296.0, 95 % CI: 74.0–1184) (data not shown).

**Table 1:** Distribution of 4492 study subjects by selected characteristics at enrolment; diagnosis of hepatocellular carcinoma (HCC) at follow-up; HCC incidence rates and hazard ratios for HCC with 95 % confidence intervals (CI). Naples, 2003–2012

Characteristics at enrolment	Study subjects	HCC cases	Incidence Rate/10^5^ (95 % CI)	Hazard Ratios (95 % CI)^b^
	No.	No.		
Total	4492	22	63.3 (41.7–96.2)	
Gender				
Female	2482	10	51.4 (27.6–95.5)	1*
Male	2010	12	78.5 (44.6–138.3)	1.2 (0.5–2.7)
Age at testing (years)				
< 60	3349	3	11.3 (3.6–35.0)	1*
60–69	551	9	214.7 (111.7–412.6)	7.5 (1.8–30.8)
≥ 70	592	10	252.8 (136.0–469.8)	6.8 (1.6–29.3)
Chronic HCV/HBV infections**				
Anti HCV-negative/HBsAg-negative	4065	2	6.3 (0.8–22.8)	1*
HCV RNA-negative/HBsAg-positive	95	3	403.3 (83.2–1179)	75.0 (12.3–456.5)
HCV RNA-positive/HBsAg-negative	239	15	909.3 (509.0–1500)	61.8 (13.3–286.4)
HCV RNA-positive/HBsAg-positive	4	0	0.0 (0.0–11,938)	0.0 (0.0-∞)
γ-glutamyl transferase (γGT) U/L^a^				
≤ 30	3588	9	32.2 (16.8–61.9)	1*
31–90	784	5	83.9 (34.9–201.6)	1.5 (0.5–4.7)
> 90	116	8	1006 (503.0–2011)	10.3 (3.6–29.2)
Alanine transaminase (ALT) U/L^a^				
≤ 40	3954	8	26.1 (13.1–52.2)	1*
41–120	490	9	240.1 (125.0–461.5)	4.1 (1.5–11.4)
> 120	45	5	1598 (665–3840)	10.3 (3.2–33.3)
Aspartate aminotransferase (AST) U/L^a^				
≤ 40	4294	6	18.0 (8.1–40.0)	1*
41–120	170	11	885.1 (490.2–1598)	9.4 (3.1–28.8)
> 120	25	5	3539 (1473–8501)	27.4 (7.6–98.0)
Platelet count (10^3^/μl)^a^				
220–700	2253	2	11.4 (1.4–41.1)	1*
< 220	2203	20	118.4 (72.3–182.8)	50.0 (1.0–100.0)
FIB-4^a^				
< 1.45	3552	1	3.6 (0.5–25.4)	1*
1.45–3.25	774	5	88.3 (36.8–212.2)	
> 3.25	130	16	1889 (1157–3083)	17.6 (6.2–49.7)

^a^The sum does not add up to the total because of missing values; ^b^adjusted for gender, age, chronic HCV/HBV infections – except FIB-4, which was adjusted for gender and chronic HCV/HBV infections since age is included in the index; *reference category; **89 HCV-positive, RNA-negative study subjects who developed 2 HCC cases were excluded

At multivariable analysis, statistically significant risk factors for HCC included age ≥60 years, HCV RNA-positivity (HR = 61.8), HBsAg-positivity (HR = 75.0), elevated values of markers of hepatic metabolic activity, with elevated risks (highest versus normal level) ranging from 10-fold for γGT to 50-fold for platelet below 220. Increasing values of FIB-4 index were associated with increasing HCC risks – e.g., the highest FIB-4 tertile was coupled with a 17.6-fold higher risk of HCC, as compared to cohort members with lower values (95 % CI: 6.2–49.7) (Table 1).

**Fig. 2:**
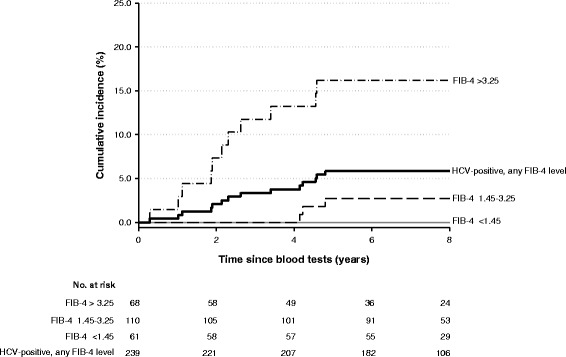
Incidence of hepatocellular carcinoma among HCV RNA-positive/HBsAg-negative subjects

The distribution of cohort members according to chronic HCV infection, FIB-4 index and HCC is described in Table 2. HCV infection and FIB-4 index were strongly associated: 59.1 % of individuals with the highest FIB-4 tertile (68/115) were chronically infected with HCV, as compared to 15.4 % (110/714) of individuals with the intermediate FIB-4 tertile, and 1.8 % for those with the lowest FIB-4 tertile. The HR of HCC directly increased with increasing values of FIB-4, in both anti HCV-negatives and in HCV RNA-positives. In anti HCV-negatives, HCC risk was 79-fold elevated (95 % CI: 4.9–1285) in those with the highest FIB-4 tertile –as compared to the lowest one. Conversely, in HCV RNA-positive cohort members, HCC risk was 771-fold higher (95 % CI: 100–5949) in those with the highest FIB-4 tertile –as compared to the lowest one (Table 2). The highest HCC cumulative incidence (17.4 % after 4 years of follow-up) was recorded among HCV RNA-positives with the highest FIB-4 tertile (Fig. 2).

**Table 2:** Hazard ratios (HR) with 95 % confidence intervals (CI) for hepatocellular carcinoma (HCC) among 4270^a^ study subjects, according to HCV RNA-positivity and FIB-4 index. Naples, 2003–2012

HCV RNA	FIB-4 <1.45			FIB-4 1.45–3.25			FIB-4 >3.25		
	Subjects No.	HCC cases No. (%)	HR^b^ (95 % CI)	Subjects No.	HCC cases No. (%)	HR^b^ (95 % CI)	Subjects No.	HCC cases No. (%)	HR^b^ (95 % CI)
Negative	3380	1 (0.03)	1*	604	0 (0.0)	0 (0-∞)	47	1 (2.1)	79.5 (4.9–1285)
Positive	61	0 (0.0)	0 (0-∞)	110	3 (2.7)	99.5 (10.3–957.3)	68	12 (17.6)	771.2 (100.0–5949)

^a^99 HBsAg-positive and 89 anti-HCV-positive, HCV-RNA-negative study subjects were excluded. Information on FIB-4 index was not available for 34 remaining study subjects

^b^Hazard ratios adjusted for gender

*Reference category

**Fig. 3:**
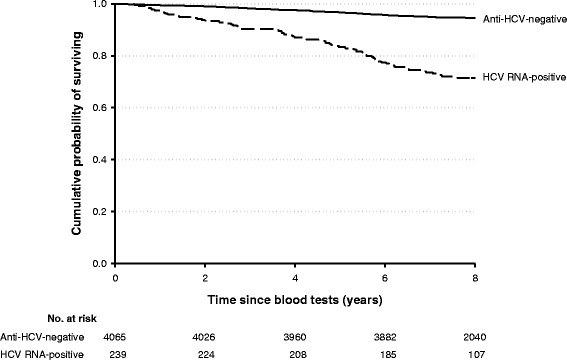
Cumulative survival probability by chronic HCV infection among 4304 study subjects. Naples, 2003–2012. * 99 HBsAg-positive and 89 HCV-positive, HCV-RNA-negative study subjects were excluded

Furthermore, HCV-infection negatively affected the survival of the cohort members: after 8 years of follow-up, 94.6 % of the 4065 HCV-negatives was alive, as compared to 71.5 % of HCV RNA-positive individuals (*p* < 0.001) (Fig. 3).

## Discussion

This population-based cohort study of individuals tested for hepatitis viruses was conducted in an area of southern Italy with one of the highest incidence rates of HCC in Europe [1]. The study findings confirmed that people chronically infected with HBV (i.e., HBsAg-positive individuals) or HCV (i.e., anti HCV RNA–positive individuals) are at a substantially higher risk of HCC than uninfected ones. In addition, to substantiate the well-established role of HCV on heightening the risk of HCC, the study findings add further, population-based evidence to the few studies that have already documented the prognostic value of the FIB-4 index as a non-invasive screening tool for HCC.

Early detection of HCC has remarkable survival advantages, and the efficacy of HCC screening is a matter of debate with particular regard to the role of non-invasive screening tools as alternative to, or in combination with, liver biopsy and fibroscan [22, 23]. A recent meta-analysis of studies carried out in patients with chronic HBV infection highlighted a modest diagnostic accuracy and sensitivity of the FIB-4 index for predicting liver fibrosis in these patients [24]. Conversely, in a cohort study involving 986 Korean HBsAg carriers, the FIB-4 index was shown to have a valuable role in HCC screening, as HBsAg carriers with high FIB-4 index were at very high risk of HCC [25]. High levels of FIB-4 were also significantly predictive of HCC among moderate or heavy alcohol drinkers [26], and among HIV-infected patients [27].

The FIB-4 index was firstly proposed to assess the presence of liver fibrosis in patients with HCV/HIV co-infection [28] and, subsequently, it was validated in a cohort of HCV-infected patients in France [15], and in a large cohort in the United States. FIB-4 was shown to be a valid parameter for staging liver disease and monitoring HCV-infected patients in longitudinal studies [16]. Since advanced fibrosis is among the most important indicators in stratifying patients with chronic HCV infection for retreatment, Tanwar and colleagues [29] compared the performance of ten biomarkers of fibrosis among patients with chronic HCV and treatment failure. They assessed the impact on biomarker performance of two different assays of hyaluronic acid. Although many biomarkers exhibited good diagnostic performance for the detection of advancing fibrosis, the study findings highlighted a potential drawback, since the diagnostic performance was significantly affected by the selection of individual component assays [29].

In the French cohort, FIB-4 values lower than 1.45 had a negative prognostic value of 94.7 % of excluding a severe fibrosis, while a value higher than 3.25 had a positive predictive value of 82.1 % [15]. Accordingly, the findings from this cohort study highlighted that individuals with a value of FIB-4 index higher than 3.25 were at a 17-fold elevated HCC risk – as compared to those with lower values of FIB-4. In particular, the highest HCC incidence was recorded among chronically HCV-infected cohort members with the highest FIB-4 tertile. An increased incidence of HCC was also noted in individuals with intermediate FIB-4 tertile, thus our findings point to a significant predictive role of FIB-4 in identifying individuals at high risk of HCC.

In the area where the present study was conducted, more than 90 % of HCC cases were attributable to HCV and/or HBV infections, and the remaining to alcohol intake (6 %), smoking or other risk factors [30]. Accordingly, only two of the 22 HCC cases documented in this cohort were not chronically infected with HCV or HBV, whereas 15 were HCV chronic carriers and HBs-Ag-negative.

In areas with high prevalence rates of HCV infection, as the one where this cohort study was carried out, the FIB-4 index of fibrosis seems to significantly improve the early identification of individuals at higher risk of developing HCC. From this perspective, it is worth noting that the time from FIB-4 measurement and HCC diagnosis decreased from 6.8 years in the HCC case with FIB-4 value <1.45, to a median of 2.8 years among HCC cases with a FIB-4 value higher than 3.25.

The rapid development of direct-acting antiviral (DAA) agents for HCV infection has driven substantial optimism on new therapeutic interventions, with regard to improved efficacy, lower frequency of side effects, and shorter duration than currently available therapies. However, the potential effect of these new therapies is limited because of lack of systematic screening for HCV infection and the high cost of DAA. Among the members of this cohort, at time of testing less than half (*n* = 149) of the 332 HCV-infected individuals was aware of being infected, and only 42 of them (28.2 %) had already received conventional interferon-based treatment. Given the costs of DAA, there is heated debate on how to prioritize patients to be treated with these drugs, with European guidelines suggesting to privilege patients with advanced liver disease for DAA therapies [31]. The findings from this study may further help in addressing this issue, by suggesting that people with chronic HCV infection with a value of FIB-4 index >3.25 are at elevated risk of developing HCC in the next 4 years. Furthermore, people with chronic HCV infection with a value of FIB-4 index ≥1.45 are at elevated risk of developing HCC in the next 5–6 years.

It is worth stressing that these results derive from about 8 years of follow-up of 4492 people living in an area of southern Italy covered by a population-based cancer registry – the CCR [32]. Completeness and accuracy in the ascertainment of HCC cases occurring in the cohort was thus ensured by the longitudinal study design. Lack of information, at baseline, on some variables known to be associated with HCC risk (e.g., alcohol intake), or at follow-up on determinants of progression of liver disease (e.g., HCV treatment) needs to be mentioned. Furthermore, a relative small number of events limited the statistical power of sub-group analysis. Moreover, it was not feasible to estimate HCC risk in HBsAg-positives and in anti HCV-positive/HCV RNA-negative individuals because of a reduced statistical power due to a small number of HCC diagnoses (*n* = 3, and *n* = 2 –respectively).

In addition to the above discussed impact of HCV, HBV and FIB-4 index of fibrosis, our findings from the multivariable analysis confirmed the prognostic indication associated with other well established predictors of HCC. Values of ALT, AST, and γGT higher than normal were indeed associated with high risks of HCC even after adjustment for HCV infection, HBV infection and FIB-4 index.

## Conclusions

Although the standard method to assess hepatic fibrosis in chronic hepatitis remains the histologic staging of liver biopsy specimen, findings from this population-based cohort study support the results of the few studies that have already documented a predictive role of FIB-4 index of fibrosis for HCC occurrence. This effect was particularly evident in people with chronic HCV infection, thus offering new insights for identifying people at high HCC risk worth to be prioritized for receiving anti-HCV treatments.

## Abbreviations

ALT: Alanine transaminase
AST: Aspartate aminotransferase
CCR: Campania Cancer Registry
CI: Confidence intervals
DAA: Direct-acting antiviral
HBV: Hepatitis B virus
HCC: Hepatocellular carcinoma
HCV: Hepatitis C virus
HR: Hazard ratios
IR: Incidence rates
PY: Person-years
UNL: Upper normal limit
γGT: Gamma-glutamyl-transferase
